# Characteristics and Microbiome Profiling of Korean Gochang Bokbunja Vinegar by the Fermentation Process

**DOI:** 10.3390/foods11203308

**Published:** 2022-10-21

**Authors:** Hoonhee Seo, Saebim Lee, Hyuna Park, Sujin Jo, Sukyung Kim, Md Abdur Rahim, Asad Ul-Haq, Indrajeet Barman, Youngkyoung Lee, Ayoung Seo, Mijung Kim, Il-yun Jung, Ho-Yeon Song

**Affiliations:** 1 Department of Microbiology and Immunology, School of Medicine, Soonchunhyang University, Cheonan-si 31151, Korea; 2 Probiotics Microbiome Convergence Center, Soonchunhyang University, Asan-si 31538, Korea; 3 Korea Balsamic Vinegar Association, Gochang 56469, Korea

**Keywords:** Gochang bokbunja vinegar, microbiome, metagenomics, Gochang vinegar, natural fermentation

## Abstract

As NGS (next-generation sequencing) technology develops, metagenomics-based microbial ecology, that is, microbiome research, has recently led to the science of fermented food. Based on the above technology, a study was conducted to understand the characteristics of vinegar made from bokbunja, a local crop in Gochang-gun, Korea. Physicochemical characteristics of vinegar, organic acid analysis, microbial community analysis, and electronic tongue analysis were explored while fermenting the vinegar for 70 days under eight fermentation conditions according to the concentration of bokbunja liquid (100% or 50%), type of fermenter (porcelain jar or stainless container), and fermentation environment (natural outdoor conditions or temperature/oxygen controlled). As a result, distinct microbial community patterns were found in the stage of acetic acid fermentation and, accordingly, this fermentation of Gochang vinegar is classified into three categories. Vinegar prepared by the traditional method of outdoor fermentation using jars showed characteristics of “*Acetobacter* (42.1%)/*Lactobacillus* (56.9%) fusion fermentation”. Under conditions where oxygen and temperature were controlled indoors using jars, characteristics of “*Komagataeibacter* (90.2%) fermentation” were found. “*Lactobacillus* (92.2%) fermentation” characteristics were discovered under natural outdoor conditions using stainless steel containers. These fermentation pattern differences were related to taxonomic phylogenetic diversity, which was also considered involved in determining organic acid production and taste. These results will be helpful as a scientific basis for understanding the fermentation characteristics of Gochang vinegar and developing high-value-added traditional vinegar products.

## 1. Introduction

Microbiome refers to an entire habitat, including microorganisms, genomes, and environmental conditions. The development of analytical platforms such as nucleic acids and metabolites combined with increased computing technology continues to expand research in the microbiome field [1]. In order to define the ecological community of commensal, symbiotic, and pathogenic microorganisms that are determinants of health and disease while sharing human body space, Lederberg has proposed a coinage called the microbiome [2]. Human microbiome research has been conducted since then. Representatively, the Human Microbiome Project [3] and MetaHIT (the European Union Project on Metagenomics of the Human Intestinal Tract) [4] have conducted extensive microbiome research on various human body organs. These extensive studies have found that the human microbiome is closely related to disease and health; hence it is called our second genome or other genome [5,6]. Moreover, the microbiome has been studied extensively concerning numerous diseases such as inflammatory bowel disease, multiple sclerosis, diabetes, allergy, asthma, autism, and cancer [7].

In addition to humans, microbiome analysis based on metagenomics has become ubiquitous in the field of ecosystem exploration, including air, soil, water, and plants, and has recently begun to be applied to food [8]. To ensure a better understanding of how to improve food quality and safety, the microbiome has been researched, particularly concerning the manufacture and maturation of traditional fermented products by category, including dairy, cereals, fish, legumes, meat, tea, and vegetables [9]. As microbial ecology studies are the basis for understanding microbial interactions that drive premium quality processes and advances in fermentation, microbiome research provides a promising opportunity to improve food quality [10].

Vinegar, used as a seasoning for pickled fruits and vegetables, mayonnaise, salad dressings, and mustard is traditionally produced from fruit juice and undergoes a microbial fermentation process based on acetic acid bacteria [11]. Vinegar contains various polyphenols, micronutrients, and other bioactive compounds which have pharmacological effects and have been used as therapeutics in many cultures [12]. In addition, various studies have been conducted on health improvement and disease treatment using vinegar. It is reported that vinegar intake positively affects infectious diseases, diabetes, cancer, and heart disease [13]. Additionally, due to vinegar’s acetic acid properties and the final product’s organoleptic properties, sophisticated process control techniques and biotechnological processes are essential for the product’s final quality [14]. According to some reports, the need for microbiome research on vinegar is growing in connection with these results [15,16,17,18,19]. However, microbiome research on vinegar is still in its infancy.

Bokbunja refers to the fruits and derivatives of Korean black raspberry (*Rubus coreanus* Miquel) and is native to Korea, Japan, and China [20]. In particular, Gochang-gun is the most representative producer of bokbunja, and the degree of specialization is very high, so it is sold in the market in the form of various foods as a specialty product [21]. In this regard, research on vinegar based on Gochang bokbunja is being conducted. The manufacturing method of bokbunja vinegar [21], its antioxidant effect [22], and clinical trials for patients with prediabetes [23] have been reported. In addition to these studies on Gochang vinegar, this study aims to establish a basis for understanding microbial interactions that lead to premium quality processes and quality improvement by applying microbial ecology research, which has been attracting attention recently as the core of the fermentation field [10]. To achieve this aim, fermentation was carried out for 70 days under 8 fermentation conditions according to the concentration of bokbunja raw materials, the type of fermenter, and the temperature/oxygen conditions. Moreover, basic physical properties, organic acid concentration, microbial community, and electronic taste were analyzed for the vinegar sampled during fermentation.

## 2. Materials and Methods

### 2.1. Fermentation of Gochang Bokbunja Vinegar

The bokbunja used in this study was a ripe bokbunja harvested in Sillim-myeon, Gochang-gun, Jeollabuk-do, Korea, on 12 June 2021. A bokbunja undiluted solution was prepared by pressing and squeezing the fruit through air compression without heating the bokbunja. At this time, the extraction efficiency was about 60–65%. If 10 kg of bokbunja was extracted, about 6–6.5 kg of the undiluted solution was obtained. On 13 June 2021, bokbunja vinegar was adjusted to about 18 degrees Brix by adding white sugar to bokbunja. Fermentation was started using Fermivin yeast as a starter. A Gochang jar (design registration 30-1093692, 27 January 2021, Korean Intellectual Property Office) for vinegar had a capacity of 30 L. It was made at Gochang Pottery in Gochang-gun using a 26-L stainless steel fermenter (STS 304 model, Vinegarmall Co., Gochang, Jeonbuk, Korea). Bokbunja vinegar was prepared under eight conditions after fermenting in Daesan-myeon, Gochang-gun, Jeollabuk-do, Korea, for 70 days from 13 June 2021 to 22 August 2021. For the first condition, an undiluted solution of juiced bokbunja was used for fermentation and the Gochang jar was kept indoors with the temperature maintained at 28~30 °C using an underwater heater (HT 55W, Haiyang, China). Oxygen was added at 1.5 m^3^/min using an oxygen generator (Vitaoxy 510, Chosun, Seoul, Korea). For the second condition, juiced bokbunja stock solution and purified water were mixed at a ratio of 5:5 and maintained following the above conditions. For the third and fourth conditions, the jar was left outdoors in its natural state without arbitrarily adjusting temperature or oxygen. The other parameters for these conditions were the same as the first and second conditions, respectively. For the fifth to eighth conditions, a stainless steel fermenter was used instead of a jar; the other criteria were the same as the first to fourth conditions, respectively. Sampling was performed 13 times from the manufacturing day (day 0) to the 1st, 3rd, 7th, 14th, 21st, 28th, 35th, 42nd, 49th, 56th, 63rd, and 70th from each condition. Samples were frozen immediately after sampling. Next, they were thawed naturally for use in this study. Abbreviations for names of conditions 1 to 8 were J/A/100%, J/A/50%, J/N/100%, J/N/50%, S/A/100%, S/A/50%, S/N/100%, and S/N/50%, respectively (where J = jar, S = stainless steel, A = adjusted temperature/oxygen, N = natural fermentation, 100% = bokbunja stock solution, and 50% = diluted bokbunja stock solution). Photos of vinegar fermentation under eight conditions are provided in Supplementary Material (Figure S1).

### 2.2. Temperature, Sugar Concentration, Alcohol Concentration, Specific Gravity, pH, and Total Acidity Measurement

The temperature was measured every 15 min using a wireless thermometer (T2, Efento, Krakow, Poland) during the entire fermentation period and recorded using a data logger. Specific gravity, pH, and sugar concentration were measured with a triple hydrometer (MRBrew, China), a pH meter (pH-8100plus, ETI, UK) and a digital refractometer (HI 96801, Hanna, Romania), respectively. The alcohol content was measured according to the “Alcoholic Analysis Regulations” of the National Tax Service of Korea, as in the previous report [24]. Total acidity was measured according to the “Vinegar Total Acid Measurement Method” of the Korean Food Standards Codex of the Ministry of Food and Drug Safety, as in the previous report [25].

### 2.3. Organic Acid Analysis

Six organic acids (tartaric acid, malic acid, lactic acid, acetic acid, citric acid, and succinic acid) were analyzed with an HPLC (1260 series, Agilent, Santa Clara, CA, USA) equipped with a YMC-Triart C18 (4.6 × 250 mm, 5 μm) column. The mobile phase was 25 mM KH_2_PO_4_ (pH 2.5) and the temperature was set to 30 °C. A 210 nm diode array detector (DAD) and a 1.0 mL/min flow rate were used. The injection volume was 20 μL. Vinegar samples were diluted 10-fold with 0.4% HCl and then filtered with 0.22 μm polyvinylidene fluoride (PVDF) membrane. Standards for six organic acids were also dissolved in 0.4% HCl and used. Concentrations of standards were set to 5, 10, 25, 50, 75, 100, 150, and 200 μg/mL. Correlation values were all over 0.9999. Standards used included L (+)-tartaric acid, 99+% (purity 100%, Thermo Fisher Scientific, Waltham, MA, USA), DL-malic acid (purity 99.2%, TCI, Tokyo, Japan), DL-lactic acid (purity 90.6%, TCI, Tokyo, Japan), acetic acid (purity 99.9%, TCI, Tokyo, Japan), citric acid (purity 99.7%, TCI, Tokyo, Japan), and succinic acid (purity 98.9%, TCI, Tokyo, Japan). The organic acid analysis was performed based on the method in the previous report, with some modifications [26,27].

### 2.4. 16S rRNA Gene-Based Metagenomic Analysis Method

First, the vinegar samples were centrifuged at 3000 rpm for 10 min and washed, repeated three times. The harvested pellet was then placed in a Lysing Matrix B tube with 0.1 mm diameter beads (MP Biomedicals, Irvine, CA, USA) and physically disrupted for 30 s with a FastPrep-24 5 G instrument (MP Biomedicals, Irvine, CA, USA). QIAamp DNA Mini Kit (Qiagen, Hilden, Germany) was used for total DNA extraction. Subsequent procedures were performed according to the protocol used in our laboratory as described in previous reports [28,29]. It consists of 16 S rRNA gene amplicon library preparation and subsequent sequencing. First, the V4 hypervariable region of the 16 S rRNA gene was amplified using a universal primer for the V4 region. Next, a metagenomic library was prepared using the Nextera XT DNA Library Prep Kit (Illumina, San Diego, CA, USA). Finally, it was mixed with PhiX control v3 (Illumina, San Diego, CA, USA), sequenced using the Illumina iSeq 100 platform (Illumina, San Diego, CA, USA), and analyzed using the EzBioCloud server (CJ Bioscience, Seoul, Korea). After all PCR steps, the product was purified using Agencourt AMPure XP magnetic beads (Beckman Coulter, Brea, CA, USA. The quality was confirmed with ChemiDoc (BioRad, Hercules, CA, USA) by agarose gel electrophoresis (Mupid, Takara, Kusatsu, Shiga, Japan). For the quantification of PCR products, the Qubit dsDNA HS Assay Kit (Thermo Fisher Scientific, Waltham, MA, USA) was used, which was measured with a Qubit Fluorometer (Thermo Fisher Scientific, Waltham, MA, USA).

### 2.5. Electronic Tongue Analysis

An ASTREE Electronic Tongue (Alpha Mos, Toulouse, France) was used for electric tongue analysis. A total of seven types of sensors were used: AHS (sourness), CTS (saltiness), NMS (umami), ANS (sweetness), SCS (bitterness), CPS (taste), and PKS (taste) [30]. These analyses were performed at Kongju University (Chungnam, Korea).

## 3. Results

### 3.1. Exploration of Basic Physicochemical Properties of Vinegar by Fermentation Condition and Period

**Figure 1:**
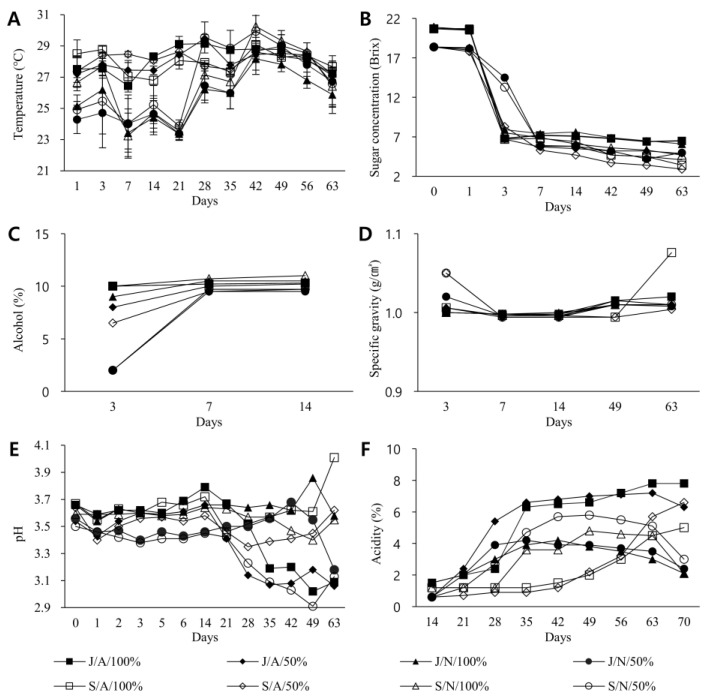
Analysis of basic physiochemical properties of vinegar by fermentation conditions and duration. During fermentation under eight different fermentation conditions, vinegar was sampled and (**A**) temperature, (**B**) sugar concentration, (**C**) alcohol concentration, (**D**) specific gravity, (**E**) pH, and (**F**) total acidity were measured. Names of the eight different fermentation conditions were: J/A/100%, J/A/50%, J/N/100%, J/N/50%, S/A/100%, S/A/50%, S/N/100%, and S/N/50% (J = fermentation in jars; S = fermentation = fermentation in stainless steel vessels). A and N represent fermentation with temperature/oxygen adjusted and natural fermentation outdoors, respectively; 100% and 50% indicate that bokbunja stock was used for fermentation, and bokbunja liquid diluted 50% with purified water was used for fermentation.

Basic physicochemical properties of vinegar, such as temperature, sugar concentration, alcohol concentration, specific gravity, pH, and total acidity by fermentation condition and fermentation time were explored. Results are shown in Figure 1.

Temperature changes were measured from the day of manufacture to the 63rd day (Figure 1A). Temperatures in the case of controlled fermentations (J/A/100%, J/A/50%, S/A/100%, and S/A/50%) were 27.5 °C, 27.2 °C, 28.5 °C, and 27.4 °C, respectively, on the starting day of fermentation. On the 63rd day, these temperatures were 26.6 °C, 27.6 °C, 26.9 °C, and 26.5 °C, respectively. On the other hand, temperatures of J/N/100%, J/N/50%, S/N/100%, and S/N/50%, fermented outdoors under natural conditions, on the day of fermentation were 25.1 °C, 24.3 °C, 26.7 °C, 24.9 °C, and 26.4 °C, respectively. On the 63rd day, they were 25.5 °C, 26.4 °C, 26.0 °C, and 26.1 °C, respectively. The average temperature was 26.5 °C, slightly lower than the group fermented by artificially controlling temperature indoors. It was measured from the lowest at 22.47 °C to the highest at 30.35 °C.

Sugar concentration was measured from the day of manufacture to the 63rd day for each fermentation condition of vinegar (Figure 1B). For vinegar conditions J/A/100%, J/N/100%, S/A/100%, and S/N/100% using bokbunja stock solution and sugar concentrations were measured to be 20.7, 20.7, 20.7, and 20.9 Brix on the day of fermentation start, respectively. They decreased sharply to 6.8, 7.9, 6.7, and 7.5 Brix on the 3rd day of fermentation, respectively. They decreased to 6.5, 6.1, 3.4, and 4.7 Brix on the 63rd day, respectively. For vinegar conditions J/A/50%, J/N/50%, S/A/50%, and S/N/50% using diluted bokbunja solution and sugar concentrations were 18.4 Brix on the day of fermentation, which was lower than those of vinegar using undiluted bokbunja solution. As with conditions using the stock solution, sugar concentrations rapidly decreased to 6.7, 14.5, 8.3, and 13.3 Brix on the 3rd day of fermentation, respectively. They decreased to 4.9, 5.0, 2.9, and 4.1 Brix on the 63rd day, respectively. Finally, the sugar concentration of vinegar fermented using undiluted bokbunja solution was higher than that fermented using diluted ones. It was also found that vinegar fermented in a jar was slightly higher in sugar concentration than in a stainless steel fermenter.

Alcohol concentration was measured three times from the 3rd day to the 14th day of fermentation (Figure 1C). Vinegar conditions J/A/100%, J/N/100%, S/A/100%, and S/N/100% fermented using undiluted bokbunja solution had alcohol concentrations of 10.0%, 9.0%, 10.0%, and 10.0%, respectively, on the 3rd of fermentation. They increased slightly to 10.3%, 10.5%, 10.3%, and 11.0% on the 14th day, respectively. For vinegar conditions J/A/50%, J/N/50%, S/A/50%, and S/N/50% fermented with diluted bokbunja liquid and alcohol concentrations were 8.0%, 2.0%, 6.5%, and 2.0% on the 3rd day, respectively. They were 10.2%, 9.5%, 9.7%, and 9.7%, respectively, on the 14th day. Alcohol concentration change was larger with diluted bokbunja solution than with undiluted stock solution of bokbunja, although the final concentration was lower with the diluted bokbunja solution. In addition, when diluted bokbunja was used, vinegar fermented in a natural state showed a slower rate of increase in alcohol concentration than those fermented with temperature/oxygen control.

Changes in specific gravity were measured from the day of manufacture to the 63rd day (Figure 1D). For vinegar conditions J/A/100%, J/N/100%, J/A/50%, and J/N/50% fermented in jars, specific gravity values were 1.080, 1.070, 1.080, and 1.070 g/cm^3^, respectively, on the starting day of fermentation. They decreased to 0.998, 0.994, 0.998, and 0.996 g/cm^3^, respectively, on 7th day. They increased again on the 63rd day, ending at 1.020, 1.010, 1.010, and 1.008 g/cm^3^, respectively. For vinegar conditions S/A/100%, S/N/100%, S/A/50%, and S/N/50% fermented in stainless steel containers, specific gravity values were 1.080, 1.070, 1.082, and 1.070 g/cm^3^, respectively, on the fermentation start date. Like vinegar fermented in jars, specific gravity values of vinegar fermented in stainless steel containers decreased to 0.996, 0.994, 0.998, and 0.994 g/cm^3^, respectively, on the 7th day. On the 63rd day, they increased more, reaching respective values of 1.076, 1.004, 1.010, and 1.010 g/cm^3^. Specifically, vinegar fermented by controlling temperature and oxygen in a stainless steel container using undiluted bokbunja solution finally had the largest specific gravity.

Changes in the pH of vinegar were measured from the day of preparation to the 63rd day (Figure 1E). For vinegar conditions J/A/100%, J/N/100%, J/A/50%, and J/N/50% fermented in jars, pH values were 3.76, 3.81, 3.76, and 3.81, respectively, on the starting day of fermentation. They were 3.55, 3.51, 4.02, and 3.66, respectively, on the 63rd day. For vinegar conditions S/A/100%, S/N/100%, S/A/50%, and S/N/50% fermented in stainless steel containers, pH values were 3.76, 3.81, 3.76, and 3.76, respectively, on the first day of fermentation. They were 4.39, 4.04, 3.93, and 3.57, respectively, on the 63rd day. Under the condition of temperature/oxygen control, it was confirmed that the pH of vinegar fermented in a jar for about 14 days decreased slightly as fermentation proceeded. In contrast, the pH of vinegar under stainless conditions increased. When fermented in the natural state, the pH of vinegar in jar condition increased. Under natural fermentation conditions using stainless steel fermenters, the pH of vinegar fermented using undiluted bokbunja solution increased, whereas vinegar using diluted bokbunja solution decreased.

Changes in total acidity were measured from the starting day of fermentation to the 70th day (Figure 1F). Changes in total acidity of vinegar conditions, J/A/100%, J/A/50%, S/A/100%, and S/A/50% fermented with temperature/oxygen control, were 0.0% on the beginning day of fermentation. They increased to 1.5%, 0.6%, 1.2%, and 0.6%, respectively, on day 14 and reached at 7.8%, 6.3%, 5.0%, and 6.6%, respectively, on day 70. Changes in total acidity of naturally fermented J/N/100%, J/N/50%, S/N/100%, and S/N/50% vinegar conditions were 0.0% on the starting day of fermentation. They increased to 1.5%, 0.6%, 1.2%, and 0.6%, respectively, on day 14, ending at 2.1%, 2.4%, 2.1%, and 3.0%, respectively, on the 70th day of fermentation. These values were lower than those of temperature/oxygen-controlled fermented vinegar.

### 3.2. Changes in Major Organic Acids by Fermentation Condition and Duration

**Figure 2:**
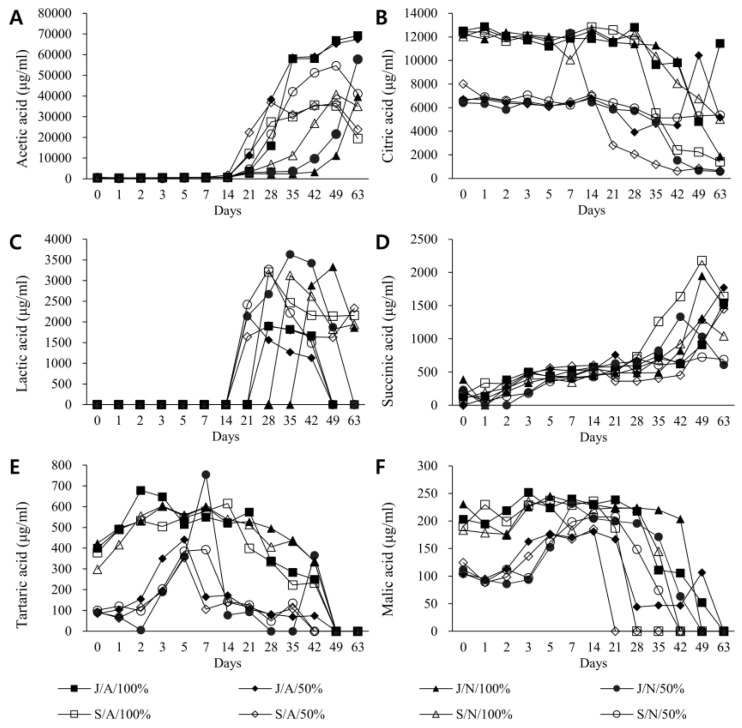
Quantitative changes of major organic acids by fermentation conditions and duration. Concentrations of (**A**) acetic acid, (**B**) citric acid, (**C**) lactic acid, (**D**) succinic acid, (**E**) tartaric acid, and (**F**) malic acid in vinegar samples over the entire period of fermentation were measured. Vinegar was fermented in Gochang jars (J) or stainless steel (S) containers. They were fermented under conditions of adjusted temperature/oxygen (A) or were fermented outdoors in a natural state (N). Two types, 100% and 50%, were used for fermentation according to concentrations of bokbunja raw materials.

Changes in major organic acids by fermentation condition and period were analyzed from the starting day of fermentation to the 63rd day of fermentation (Figure 2). Acetic acid was the highest among measured organic acids (Figure 2A). For J/A/100%, J/N/100%, J/A/50%, and J/N/50% vinegar conditions fermented with jars, acetic acid concentrations were 399, 420, 747, and 526 μg/mL on the beginning day of fermentation, respectively. Starting on the 21st day, they increased sharply to 69,299, 67,434, 39,731, and 578,506 μg/mL, respectively. For S/A/100%, S/N/100%, S/A/50%, and S/N/50% vinegar conditions fermented in stainless steel containers, acetic acid concentrations were 512, 405, 431, and 6096 μg/mL, respectively, on the starting day of fermentation. Like those fermented in jars, acetic acid in the vinegar in stainless steel fermenters also increased rapidly on the 21st day, peaking on the 42nd to 49th days. They decreased, ending at 19,454, 23,946, 35,049, and 411,396 μg/mL, respectively, on the 63rd day of fermentation.

Citric acid accounted for the highest proportion of vinegar after acetic acid (Figure 2B). For vinegar conditions J/A/100%, J/N/100%, S/A/100%, and S/N/100% fermented with undiluted bokbunja solution, citric acid concentrations were 12,508, 12,236, 12,436, and 12,051 μg/mL on the start day, respectively. They decreased to 11,446, 1884, 1424, and 5019 μg/mL, respectively, on the 63rd day. For vinegar conditions J/A/50%, J/N/50%, S/A/50%, and S/N/50% fermented with diluted bokbunja solution, citric acid concentrations were 6724, 6402, 8018, and 6577 μg/mL, respectively, on the start day. They decreased to 5173, 594, 657, and 5382 μg/mL on the 63rd day, respectively. Citric acid contents in vinegar fermented with undiluted bokbunja solution were generally higher than those in vinegar fermented using diluted bokbunja solution.

Among major organic acids, lactic acid had the third highest concentration in vinegar. Its concentrations increased rapidly from 3–4 weeks of fermentation under all conditions. They then showed a tendency to decrease later (Figure 2C). They were almost absent in vinegar fermented with undiluted bokbunja on the day of fermentation. However, on the 28th day under temperature/oxygen-controlled conditions, their concentrations in J/A/100% and S/A/100% increased to 1895 and 3205 μg/mL, respectively, although they were still not detected in naturally fermented vinegar. At the endpoint, their concentrations in vinegar fermented with temperature/oxygen-controlled conditions decreased but increased to about 2000 μg/mL in naturally fermented vinegar. For vinegar using 50% bokbunja liquid, lactic acid was almost absent at the start but increased to 1642–2419 μg/mL after 3 weeks and decreased on the 63rd day to 2331 μg/mL. Moreover, on the last day, 63, the concentration was maintained only in oxygen/temperature-controlled vinegar in a stainless steel barrel. However, it was not detected in the rest of the conditions.

Among major organic acids, malic acid had the fourth highest concentration in vinegar. It tended to increase as fermentation time passed in all conditions (Figure 2D). Succinic acid and malic acid in vinegar fermented using undiluted bokbunja solution maintained higher concentrations than in vinegar fermented using diluted bokbunja solution during early and middle fermentation periods. They decreased significantly to shallow levels in the late period (Figure 2E,F).

### 3.3. Changes in Composition and Diversity of Microbiome of Vinegar According to Fermentation Conditions over Time

**Figure 3:**
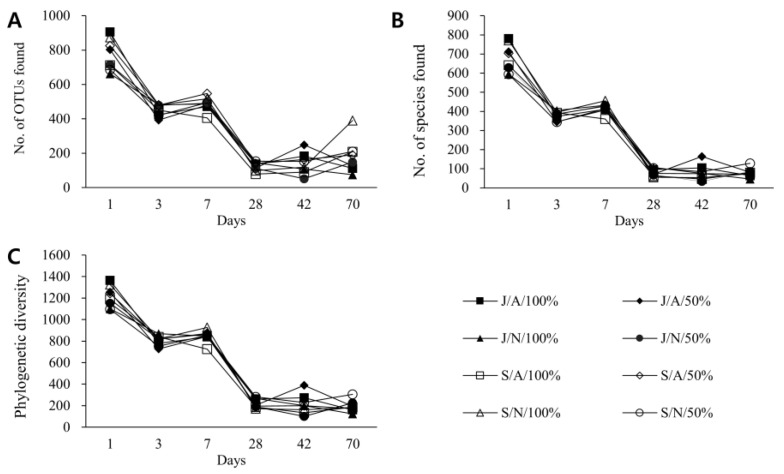
Analysis of diversity of bacterial communities in vinegar according to fermentation time and condition. As fermentation progressed, vinegar was sampled and DNA was extracted. The bacterial 16S rRNA gene was amplified, sequenced with NGS (Next-generation sequencing), and analyzed. (**A**) Sequences less than 97% similarity to other sequences were analyzed as different operational taxonomic units (OTUs). (**B**) Among them, the number of unique species identified based on the reference database was examined. (**C**) Measures of biodiversity incorporating phylogenetic differences between species were also studied. Please see the “Materials and Methods” Section for eight different fermentation conditions. For example, the first condition, ‘J/A/100%’, means that 100% bokbunja undiluted solution is fermented in a Gochang jar with oxygen/temperature adjusted.

The diversity of bacterial communities by fermentation time and condition was analyzed (Figure 3). On the first day, the average diversity of bacterial communities was 773 OTUs (operational taxonomic units). However, as fermentation progressed, it gradually decreased to an average of 183 OTUs on the 70th day (Figure 3A). An average of 678 species and 79 species was found on the 1st day and 70th day of fermentation, respectively (Figure 3B). As fermentation progressed, unknown bacteria in Gochang vinegar gradually increased from 95 species (about 12%) on the first day to 104 species (about 57%) on the 70th day. As fermentation progressed, the phylogenetic diversity index decreased and a specific bacterial species became dominant (Figure 3C). It is interesting to note that while fermenting naturally with 50% bokbunja solution in any fermentation vessel, the phylogenetic diversity index on day 70 was about 1.7–1.8 times higher than that of the stock solution. Additionally, under natural fermentation conditions with 50% bokbunja liquid, the phylogenetic diversity index was higher than it was under oxygen/temperature-controlled conditions, which were about 1.2 times when fermented in a jar and 1.8 times when fermented in a stainless steel container.

**Figure 4:**
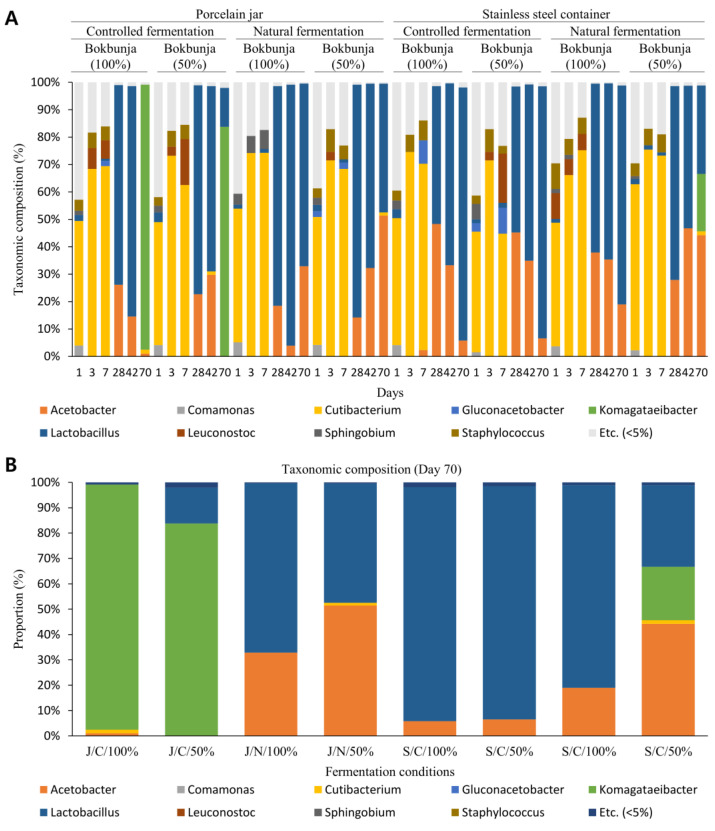
Changes in the taxonomic composition according to fermentation condition and duration. (**A**) After sampling while fermenting vinegar for each fermentation condition, 16S rRNA gene-based metagenomics analysis was performed. Under all conditions, *Cutibacterium* in the early stage of fermentation and *Lactobacillus* in the middle of fermentation showed a common dominance tendency. (**B**) However, in the late fermentation period, three fermentation patterns were observed. Vinegar fermented under temperature/oxygen-controlled conditions in jars showed *Komagataeibacter* fermentation characteristics. Moreover, vinegar naturally fermented in jars showed complex fermentation characteristics of *Lactobacillus* and *Acetobacter*. On the other hand, vinegar fermented under temperature/oxygen-controlled conditions in stainless steel containers has characteristics of *Lactobacillus* fermentation. Other vinegars showed a fusion form of the above three types.

Changes in microbiome composition in vinegar were analyzed over time for each fermentation condition (Figure 4A). Under all fermentation conditions, *Cutibacterium* was dominant as it gradually increased until the first week of fermentation. The composition ratio was 44.0–60.6% on the 1st day, 66.2–75.5% on the 3rd day, and 44.8–75.2% on the 7th day. It showed that *Lactobacillus* occupied the highest composition at around 4–6 weeks, which was the middle stage of fermentation. At week 10, the end of the fermentation, several different patterns were observed depending on fermentation conditions (Figure 4B). First, results showed the absolute dominance of *Lactobacillus* in vinegar under oxygen/temperature-controlled conditions in stainless steel containers. However, natural vinegar in pots showed dominance by *Lactobacillus* and *Acetobacter* taxa. Interestingly, *Komagataeibacter* showed dominance during fermentation in the jar that was oxygen/temperature controlled. On the other hand, fermentation in a natural state in a stainless steel container resulted in a mixture of the above three forms. Using an undiluted bokbunja solution, *Lactobacillus*, partially composed of *Acetobacter*, was dominant. Using 50% bokbunja solution, *Lactobacillus*, *Acetobacter*, and *Komagataeibacter* were evenly dominant.

### 3.4. Electronic Tongue-Based Taste Pattern Analysis

**Figure 5:**
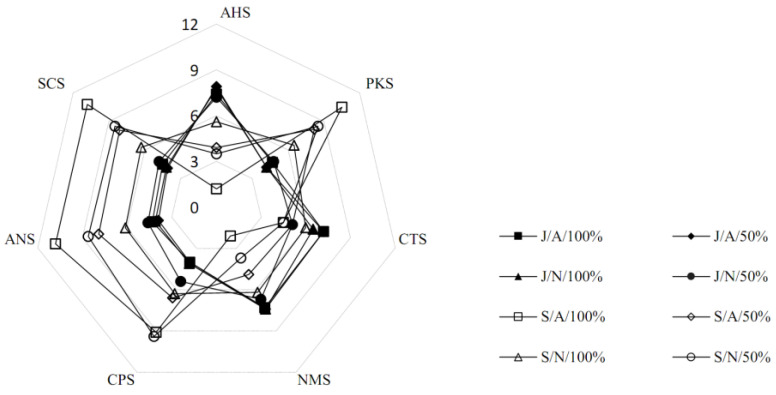
Comparative analysis of electronic tongue-based taste patterns in vinegar. According to fermentation conditions, the taste pattern of bokbunja vinegar was analyzed using an electronic tongue. Seven types of sensors were used for the analysis: AHS indicates sour taste, CTS indicates salty taste, NMS indicates umami, ANS indicates sweet taste, and SCS indicates bitter taste. It was analyzed that vinegar fermented in a jar had a sour and umami taste, while vinegar fermented in a stainless steel container had a bitter and sweet taste.

According to fermentation conditions, taste patterns of bokbunja vinegar were analyzed using an electronic tongue (Figure 5). For vinegar fermented in jars, sour and umami taste were high, whereas saltiness and bitterness were low. In the case of salty taste, the detection was lower in the vinegar fermented with 50% bokbunja solution, while it was prominently tasted in the vinegar fermented with undiluted bokbunja solution. However, even with a high salty taste, naturally fermented vinegar had a lower salty taste than a temperature/oxygen-controlled one. On the other hand, vinegar fermented in stainless steel containers showed low sour and umami taste but high bitterness and sweetness. The salty taste was slightly lower at a level similar to that of fermented vinegar in a jar.

## 4. Discussion

In this study, the microbiome characteristics according to fermentation conditions were studied in the vinegar manufacturing process using bokbunja harvested in Gochang, Korea. Changes in basic physicochemical properties, the concentration of organic acids, microbial community, and taste characteristics were analyzed during the fermentation process under eight types of fermentation conditions according to the concentration of bokbunja liquid (100% or 50%) used, the type of container (porcelain jar or stainless container), and the fermentation environment conditions (outdoor natural fermentation or oxygen/temperature controlled). This study’s representative research results confirmed that Gochang bokbunja vinegar could be classified into three types based on microbiome pattern characteristics according to the manufacturing process method.

Vinegar is generally manufactured in a three-step process: starch saccharification, the conversion of sugars to ethanol by yeast, and, finally, the oxidation of ethanol to acetic acid by acetic acid bacteria; while the microbial diversity and their metabolites in this process determine the quality of vinegar, so the characteristics of the microbiome involved in fermentation are essential [31]. In the results of this study, similar microbiome profiling characteristics were shown under all conditions during the early and middle stages of fermentation. However, there was a distinct pattern difference in the late stages. Accordingly, Gochang vinegar was classified into three types, as mentioned above. Specifically, when vinegar was made by the traditional method of outdoor fermentation using a jar, it underwent “*Acetobacter*/*Lactobacillus* fusion fermentation”. Under conditions where oxygen/temperature was controlled indoors using a jar, it underwent “*Komagataeibacter* fermentation”. When it was manufactured outdoors in a natural state using a stainless steel container, it underwent “*Lactobacillus* fermentation”.

Acetic acid bacteria, a member of the genus *Acetobacter* characterized by the ability to convert ethyl alcohol to acetic acid by oxidation, is well-known as a vinegar bacterium and reported as the basis of many kinds of vinegar [32]. Metagenomics analysis of Zhenjiang aromatic vinegar in China has revealed that *Acetobacter* eventually dominates in the late fermentation period, supporting the existing theory [16]. This vinegar was fermented with MSSF (multispecies solid-state fermentation) and *Lactobacillus* was dominant in the early fermentation stage, whereas *Acetobacter* was dominant in the latter period. The results also showed that bacteria contributed more to flavor formation than fungi, highlighting the importance of characterizing the bacterial community in acetic acid fermentation. On the other hand, in Gochang vinegar, the absolute dominance of *Acetobacter* was not observed in any of the fermentation conditions tested. Under the condition of natural fermentation using a traditional vinegar jar, *Acetobacter* and *Lactobacillus* showed a fusion fermentation form, occupying half equally. In this fermentation condition, *Lactobacillus* and *Acetobacter* accounted for 56.9% and 42.1% of the total, respectively, at the end of fermentation and the two genera accounted for 99% of the total. These acetic acid bacteria, represented by *Acetobacter*, are used in industrial vinegar production, whereas Gochang vinegar is considered unique and differentiating in this respect [33].

It is known that a large number of lactic acid bacteria in vinegar can produce many lactic acids, amino acids, and other flavor compounds during vinegar fermentation [17]. Therefore, it is judged as a positive specificity that *Lactobacillus*, a representative lactic acid bacterium, was detected on par with *Acetobacter* in Gochang vinegar naturally fermented in a pot. In addition, it has been reported that *Lactobacillus* can inhibit the formation of glycation end-products (AGEs) known to cause pro-aging inflammation, nephropathy, protein denaturation, and oxidative stress during vinegar fermentation, supporting the above positive uniqueness [34]. In the case of Tianjin duliu mature vinegar, a famous traditional Chinese vinegar, a metagenomics analysis study has reported that the content of lactic acid bacteria in the whole acetic acid fermentation process is substantial and has a significant effect on the taste of vinegar [17]. According to the paper, *Lactobacillus* was maintained at around 80% from 94.7% on the first day of fermentation to 93.2% on the 24th day, the last day of fermentation. The pH at this time is reported to fluctuate between 3.7 and 3.9. In the case of Gochang vinegar, the composition of *Lactobacillus* was 92.2% in vinegar fermented under oxygen/temperature control in a stainless steel container. Characteristics dominated by *Lactobacillus* are similar to the microbiome pattern of Tianjin vinegar. However, in the case of Tianjin vinegar, *Lactobacillus* was dominant from the beginning to the end of fermentation. In contrast, in Gochang vinegar, *Acetobacter* gradually decreased from the middle of fermentation and *Lactobacillus* gradually increased under the above condition, eventually leading to dominance. Moreover, at this time, the lactic acid concentration was the highest in late fermentation compared to other fermentation conditions. However, it is worth noting that excessive growth of lactic acid bacteria is undesirable as it causes sugar loss and consequently affects the final product’s flavor [18]. Therefore, if this method is considered in the production of Gochang Bokbunja vinegar, appropriate measures may be necessary to lower the ratio of lactic acid bacteria.

Another type of Gochang vinegar that stood out was *Komagataeibacter* fermentation vinegar. Known for its high acetic acid resistance, *Komagataeibacter* is involved in industrial vinegar production [35]. When acetic acid concentrations reach 7–8%, damage occurs, while *Acetobacter* can resist 15–20% acetic acid, in which BCAAs (branched-chain amino acids) are known to play a role in its acid resistance [36]. The dominance of *Komagataeibacter* over the microbiome of European wine and organic apple cider vinegar based on next-generation sequencing has been reported [19]. In red wine vinegar, *Komagataeibacter* accounted for almost the majority, but in organic apple cider vinegar, *Komagataeibacter* was equally dominant with *Acetobacter*, followed by *Lactobacillus* and *Oenococcus*.

In this study, when fermented with constant oxygen/temperature in a jar, *Komagataeibacter* was dominant at the end of fermentation by 90.2%. This result is believed to be related to high total acidity, high acetic acid concentration, and low pH in late fermentation under the above conditions.

Although it was not analyzed in the same way at the same time, relatively much more bacteria might have participated in the fermentation of Gochang vinegar based on information reported in existing studies [16,18]. For Zhenjiang aromatic vinegar, 253 OTUs were observed during the fermentation process for 18 days [16]; 548, 268, and 153 OTUs were observed for vinegar pei on the 3rd, 18th, and 24th days of fermentation [18]. In this study, 662 to 905 OTUs were observed on day 1 and 75 to 391 OTUs on day 70, depending on the fermentation conditions. Of course, as inferred from a decrease in the diversity index as the acetic acid fermentation process as late fermentation process progressed, it seemed that particular species would dominate and exhibit some fermentation patterns. As the average diversity index was higher in all conditions in the case of using 50% dilution than the condition in which the stock solution was used, it could be used as primary data for determining the appropriate initial concentration of bokbunja and the resulting cost estimation. In addition, the average value under all conditions was higher in outdoor natural fermentation than in an artificial oxygen/temperature-controlled environment in the same 50% bokbunja liquor fermentation, which supports the appropriateness of the traditional method of vinegar production. These microbiome characteristics seem to contribute to the formation of complex organic acids. In particular, unknown bacteria were analyzed in about half during late fermentation, contributing to the specialty of Gochang vinegar. It has been confirmed that each fermentation condition’s microbiome characteristics affect the ingredients of vinegar and the taste in the end.

Although microbiome technology has reached the field of fermented food and is producing significant results, it is still in its infancy compared to the field of the human microbiome. However, in recent years, shotgun metagenomics analysis has been performed on fermented foods [37,38], and further, it has begun to be applied to metabolomics [39] and metaproteomics [40,41]. Finally, research on the microbiome of fermented foods is focused on multi-omics technology that combines the above omics [42]. Accordingly, this study is considered to be the beginning of the vinegar microbiome study and is considered necessary to reach the multi-omics level for a fundamental understanding of vinegar fermentation in the future.

## 5. Conclusions

In this study, the microbial community characteristics of Korean Gochang bokbunja vinegar according to various fermentation conditions were analyzed (Summary of this study, Figure S2). As a result, a distinct microbial community pattern was found in the acetic acid fermentation stage, primarily classified into three categories: “*Acetobacter*/*Lactobacillus* fusion fermentation”, “*Lactobacillus* fermentation”, and “*Komagataeibacter* fermentation”. These fermentation types were related to taxonomic phylogenetic diversity and, thus, organic acid production and taste. The above results are expected to be the basis for understanding the characteristics of Gochang vinegar fermentation and developing high-value-added products. However, this study has several limitations, such as narrow fermentation conditions, lack of comparison with domestic and foreign vinegar, and analysis limited to bacteria. Nevertheless, it is hoped that the results of this study can be used as a basis for microbiological studies of fermented foods beyond vinegar.

## Data Availability

The data presented in this study are available on request from the corresponding author.

## Supplementary Materials

The following supporting information can be downloaded at: https://www.mdpi.com/article/10.3390/foods11203308/s1. Figure S1: Photos of vinegar fermentation under eight conditions. In this study, vinegar was fermented under 8 different conditions for 70 days using bokbunja in Gochang-gun. In the first condition, bokbunja juice was fermented using a vinegar jar under conditions where oxygen and temperature were controlled. The second condition uses a 50% dilution made by mixing purified water in a ratio of 5:5 instead of using 100% bokbunja juice of the first condition. Unlike the first and second conditions, the third and fourth conditions were fermented outdoors in a natural state without oxygen/thermostat, respectively. The fifth to eighth conditions were identical to the first to fourth conditions described above, except that a stainless steel container was used instead of a jar. Abbreviations for names of conditions 1 to 8 were J/A/100%, J/A/50%, J/N/100%, J/N/50%, S/A/100%, S/A/50%, S/N/100%, and S/N/50%, respectively (where J = jar, S = stainless steel, A = adjusted temperature/oxygen, N = natural fermentation, 100% = bokbunja stock solution, and 50% = diluted bokbunja stock solution); Figure S2: Summary of this study (graphical abstract). Distinct microbial community patterns were found in the stage of acetic acid fermentation, and accordingly, this fermentation of Gochang vinegar is classified into three categories. Vinegar prepared by the traditional method of outdoor fermentation using jars showed characteristics of ‘*Acetobacter* (42.1%)/*Lactobacillus* (56.9%) fusion fermentation’. Under conditions where oxygen and temperature were controlled indoors using jars, characteristics of ‘*Komagataeibacter* (90.2%) fermentation’ were found. ‘*Lactobacillus* (92.2%) fermentation’ characteristics were discovered under natural outdoor conditions using stainless steel containers. The average taxonomic composition ratio is the average of the values under two concentration conditions of the bokbunja stock solution.
