# Supplementary material for: Characteristics and Microbiome Profiling of Korean Gochang Bokbunja Vinegar by the Fermentation Process
Source: Foods. 2022 Oct 21;11(20):3308. doi: 10.3390/foods11203308 (PMC9601284; doi:10.3390/foods11203308)
Supplement: Supplementary file 1 [file foods-11-03308-s001.zip › foods-1963473-supplementary.pdf]

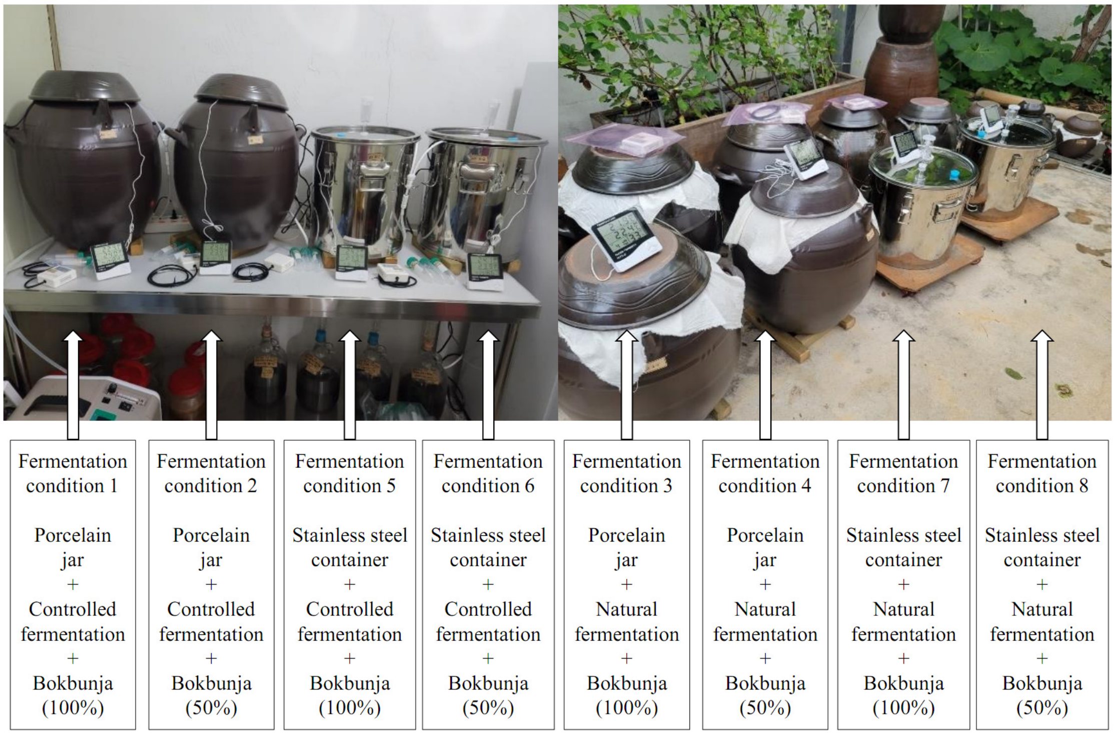

Figure S1. Photos of vinegar fermentation under eight conditions. In this study, vinegar was fermented under 8 different conditions for 70 days using bokbunja in Gochang-gun. In the first condition, bokbunja juice was fermented using a vinegar jar under conditions where oxygen and temperature were controlled. The second condition uses a 50% dilution made by mixing purified water in a ratio of 5:5 instead of using 100% bokbunja juice of the first condition. Unlike the first and second conditions, the third and fourth conditions were fermented outdoors in a natural state without oxygen/thermostat, respectively. The fifth to eighth conditions were identical to the first to fourth conditions described above, except that a stainless steel container was used instead of a jar. Abbreviations for names of conditions 1 to 8 were J/A/100%, J/A/50%, J/N/100%, J/N/50%, S/A/100%, S/A/50%, S/N/100%, and S/N/50%, respectively (where J = jar, S = stainless steel, A = adjusted temperature/oxygen, N = natural fermentation, 100% = bokbunja stock solution, and 50% = diluted bokbunja stock solution).

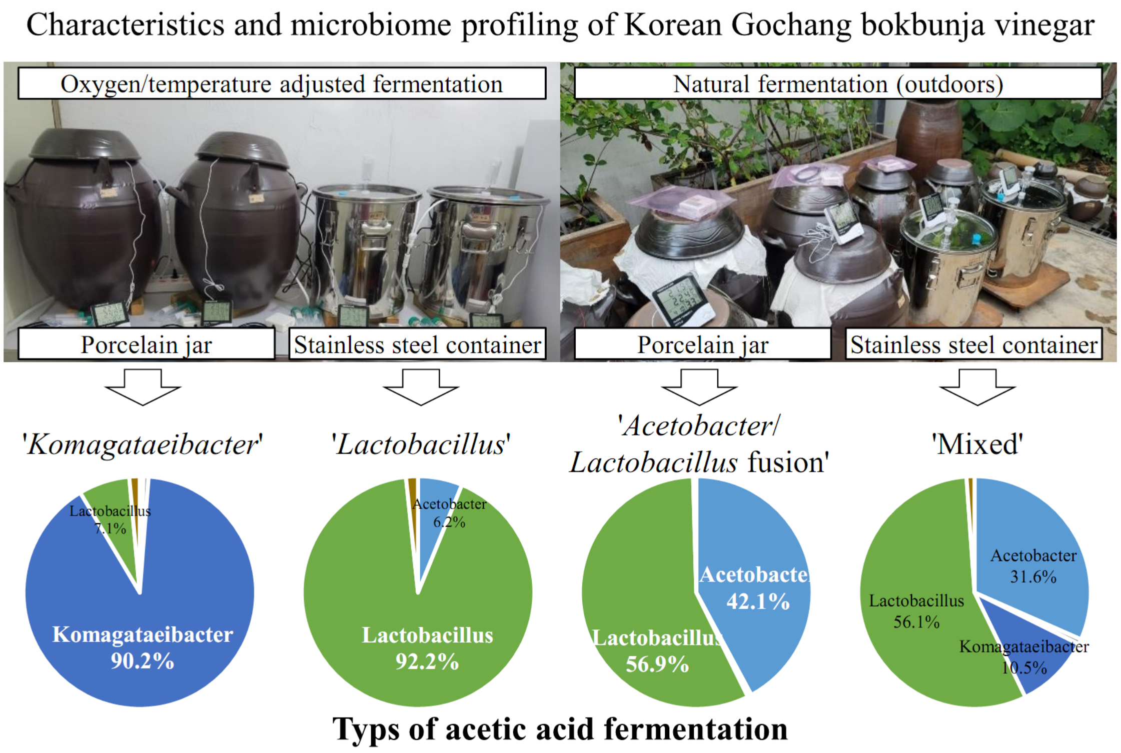

Figure S2. Summary of this study (graphical abstract). Distinct microbial community patterns were found in the stage of acetic acid fermentation, and accordingly, this fermentation of Gochang vinegar is classified into three categories. Vinegar prepared by the traditional method of outdoor fermentation using jars showed characteristics of '*Acetobacter* (42.1%)/*Lactobacillus* (56.9%) fusion fermentation'. Under conditions where oxygen and temperature were controlled indoors using jars, characteristics of '*Komagataeibacter* (90.2%) fermentation' were found. '*Lactobacillus* (92.2%) fermentation' characteristics were discovered under natural outdoor conditions using stainless steel containers. The average taxonomic composition ratio is the average of the values under two concentration conditions of the bokbunja stock solution.
